# Chronic Osteomyelitis of the Distal Femur Treated with Resection and Delayed Endoprosthetic Reconstruction: A Report of Three Cases

**DOI:** 10.1155/2017/5141032

**Published:** 2017-08-15

**Authors:** Sean Ryan, William Eward, Brian Brigman, Robert Zura

**Affiliations:** ^1^Duke University Hospital, 1308 Mallory Lane, Durham, NC 27713, USA; ^2^Duke University Hospital, 20 Duke Medicine Circle, Durham, NC 27710, USA; ^3^Louisiana State University, 1542 Tulane Avenue, Box T6-7, New Orleans, LA 70112, USA

## Abstract

Chronic osteomyelitis involving the distal femur often results in amputation or arthrodesis. This article presents three cases of chronic osteomyelitis treated with a staged approach culminating in endoprosthetic reconstruction. Stage one involved resection of infected bone and placement of an intramedullary nail spanning the bony defect between proximal femur and tibia, with antibiotic cement packed around the nail. Patients were then placed on long-term IV +/− oral antibiotics to clear the infection. A “cooldown” period was then used between stages where patients were off antibiotics and inflammatory markers were monitored for signs of remaining infection. Stage two then involved reconstruction of the distal femur and knee with an endoprosthesis. In the appropriate patient, this treatment strategy offers another option in this challenging population.

## 1. Introduction

The management of chronic osteomyelitis of the distal femur is difficult and often results in limb loss. Antibiotics are essential to treatment; however, they are often unsuccessful when used without adequate debridement of infected bone [1, 2]. Frequently, these patients have a history of fracture, and early infection progresses to chronic osteomyelitis [1, 3, 4]. The most common infecting organism is* Staphylococcus aureus* [3, 4]. Surgery is the mainstay of treatment including local irrigation and debridement, wide surgical resection, or amputation [1, 3–5]. Reconstruction following surgical intervention is often difficult, as insertion of hardware to enhance bony stability is avoided in an effort to allow subsequent antibiotic clearance of residual infection in the absence of hardware, given the risk for biofilm creation. The purpose of this paper is to present three cases of chronic osteomyelitis of the distal femur, refractory to prior debridement, treated with wide resection, placement of an antibiotic cement spacer around an intramedullary nail, subsequent IV antibiotics, and endoprosthetic reconstruction in order to spare patients the morbidity of above-knee amputations. We present these cases as a reasonable treatment strategy for distal femur osteomyelitis.

## 2. Case Report

### 2.1. Case  1

A 41-year-old male with history of diabetes and tobacco use presented with right thigh pain from distal femoral osteomyelitis secondary to presumed hematogenous dissemination of MSSA. He had previously undergone two local irrigation and debridement procedures followed by intravenous (IV) antibiotics and was advised to have a transfemoral amputation. He presented for a second opinion with a desire to avoid amputation. On his initial evaluation, he had a mild knee effusion, quadriceps atrophy, and knee range of motion 5–110 degrees. Incisions were well-healed.

**Figure 1:**
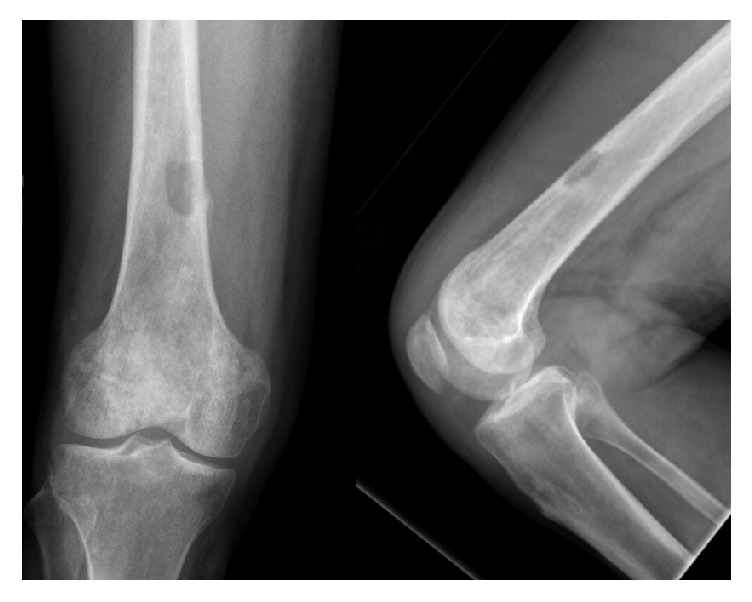
AP and lateral right knee with distal femur sequestrum/involucrum and small periosteal reaction.

**Figure 2:**
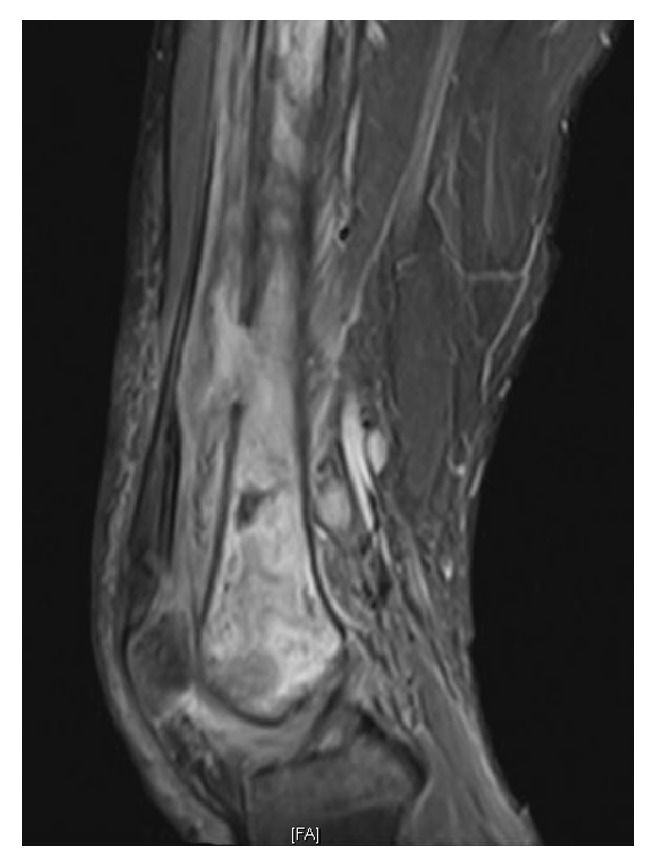
Sagittal MRI of right knee with chronic osteomyelitis from distal femur diaphysis to most distal aspect.

**Figure 3:**
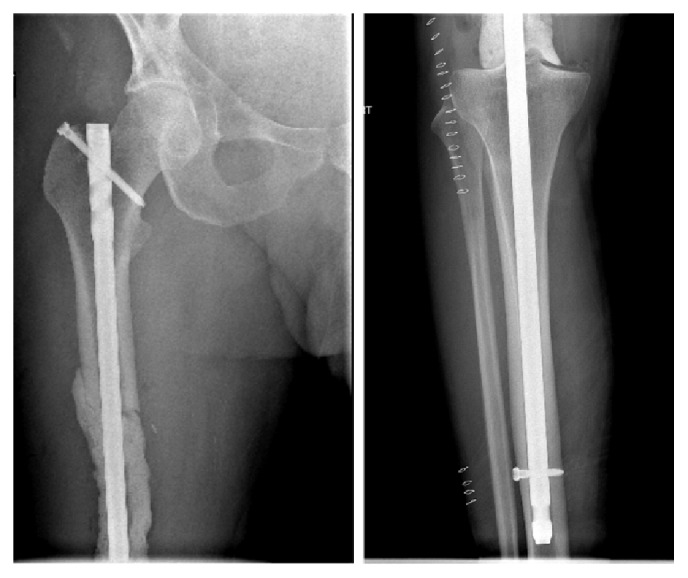
Postoperative AP radiographs showing long nail with surrounding cement.

Radiographs and MR images at presentation are shown (Figures 1 and 2) and consistent with chronic osteomyelitis. Erythrocyte sedimentation rate (ESR) and c-reactive protein (CRP) were 30 mm/hr and 0.39 mg/l, respectively. He was subsequently taken to the operating room for biopsy and excision of the distal 24 cm of the femur. The remaining femur and tibia were reamed and an 11.5 × 700 mm Smith and Nephew intramedullary nail was placed. Vancomycin cement was then used to coat the nail and the patient had primary closure of their wound. Postoperative radiographs are shown (Figure 3).

Operative cultures grew coagulase-negative* Staphylococcus* and he was treated with IV Cefazolin for six weeks, followed by six weeks of oral Cephalexin. ESR and CRP were monitored for several months following discontinuation of antibiotics during a “cooldown” period and were observed to decrease over time, reaching a low of ESR 6.0 mm/hr and CRP 0.14 mg/l.

**Figure 4:**
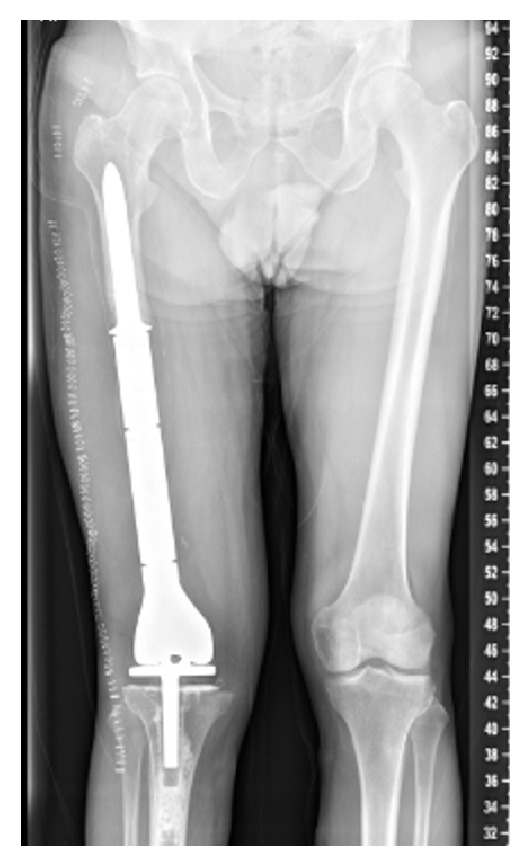
Postoperative AP radiograph with endoprosthesis reconstruction.

Eight months following placement of the intramedullary nail, once it was felt that his infection was cleared, he returned for planned reconstruction. Antibiotic cement and hardware were removed and frozen sections obtained intraoperatively showed no evidence of acute inflammation. Subsequently, a large Stryker GMRS distal femoral endoprosthesis with a rotating hinge knee was used for reconstruction. Postoperative radiographs are shown (Figure 4). He was last seen two years postoperatively and doing well with knee range of motion 0–110 degrees. He ambulates without an assistive device.

### 2.2. Case  2

A 33-year-old female smoker presented with left thigh pain attributed to distal femur osteomyelitis secondary to prior internal fixation of an open distal femoral fracture. She had two prior irrigation and debridement procedures, six weeks of IV Zosyn, and one year of oral Augmentin. She had persistent pain and the referring institution recommended above-knee amputation. At presentation, her incisions were healed; she had a knee extension lag of 20° and knee flexion to 90°. Her inflammatory markers were elevated with ESR of 56 mm/hr and CRP of 1.18 mg/l.

**Figure 5:**
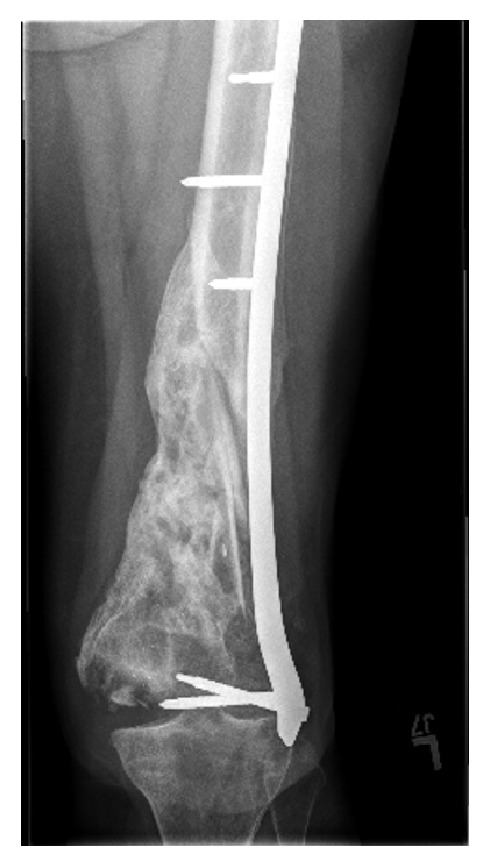
AP radiograph of distal femur.

**Figure 6:**
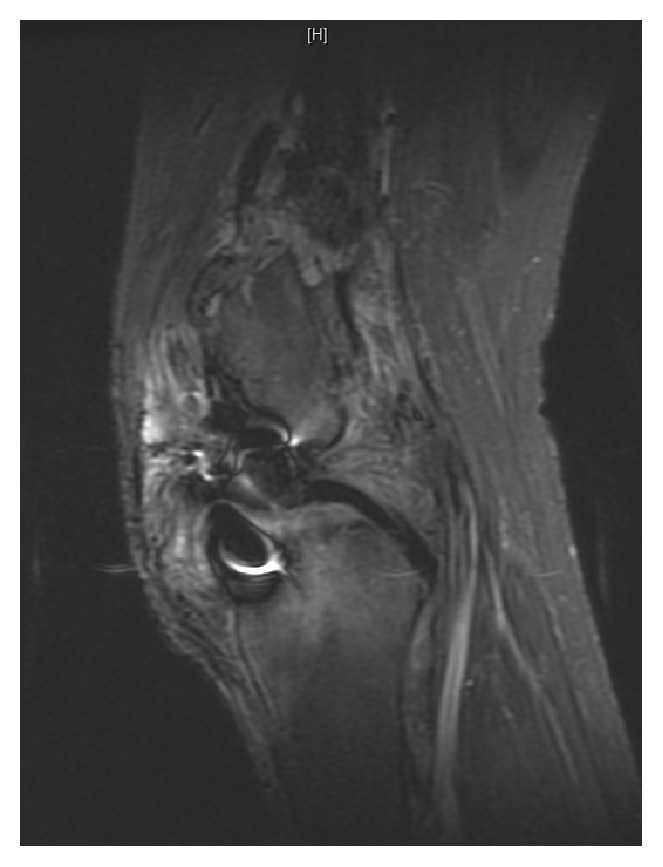
Sagittal STIR MRI of left knee with fracture nonunion of distal femur, marked synovitis, and bony changes consistent with osteomyelitis versus reactive changes.

**Figure 7:**
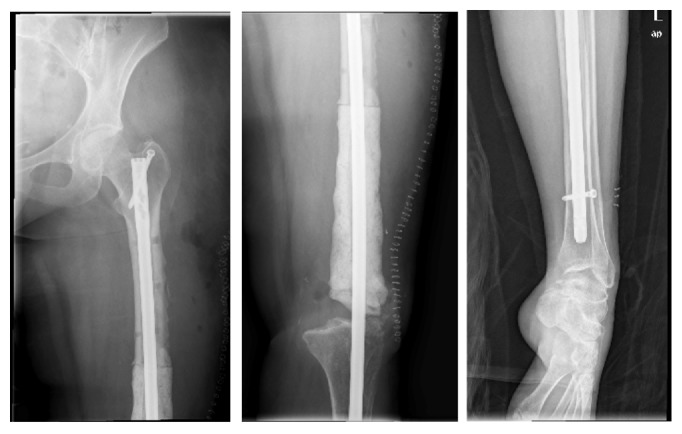
Postoperative radiographs with long IM nail and surrounding bone cement.

Radiographs and MR images at presentation (Figures 5 and 6) are shown and consistent with chronic osteomyelitis. She was taken to the operating room for bone biopsy/culture and operative cultures grew* Enterococcus* in broth. She subsequently returned to the operating room for resection of the most distal 19 cm of the femur. Intraoperatively, purulent exudate was noted in the knee and along the hardware. Her remaining proximal femur and tibia were reamed and a 12.5 × 700 mm Smith and Nephew intramedullary nail was placed. Gentamicin cement was packed around the nail and postoperative radiographs are shown (Figure 7).

Intraoperative cultures grew ampicillin-sensitive* Enterococcus*, and she completed six weeks of IV Ampicillin, followed by six weeks of oral Amoxicillin. ESR and CRP were persistently elevated, and with the assistance of infectious disease, it was decided to continue oral Amoxicillin for a total of six months. Subsequently, antibiotics were discontinued and inflammatory markers were trended during a “cooldown” period and observed to decrease over time.

**Figure 8:**
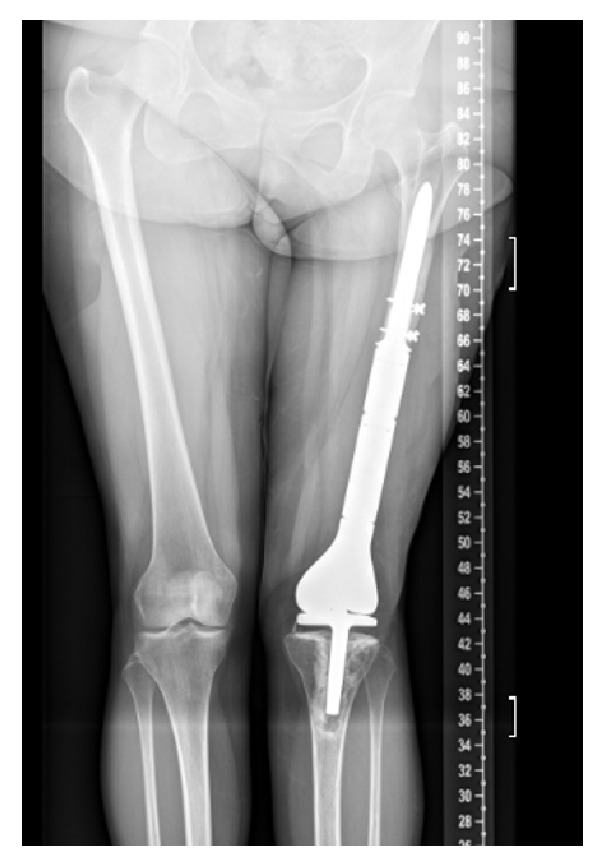
Postoperative AP radiograph with endoprosthesis reconstruction.

Five months following discontinuation her antibiotic therapy, the assessment was made that her infection had been cleared and she was taken to the operating room for planned reconstruction. Antibiotic cement and hardware were removed, and intraoperative frozen sections were negative for acute inflammation. A large Stryker GMRS distal femoral endoprosthesis with a rotating hinge knee was placed and her patellar tendon was found to be partially avulsed from the tibial tubercle. A tubularized polypropylene mesh was secured into a proximal tibial tunnel with polymethyl methacrylate and then woven into the extensor mechanism as an augment. Postoperative radiographs are shown (Figure 8). Her left leg was 4 cm shorter than the right and shoe lift orthotic provided partial correction. She was last seen two years postoperatively and is doing well with knee range of motion from 0 to 105 degrees. She was satisfied with her current status and did not desire surgical correction of leg length discrepancy.

### 2.3. Case  3

A 64-year-old female, with chronic kidney disease, hypertension, diabetes mellitus, obesity, and prior right femoral neck fracture treated with dynamic hip screw, presented with a chronic right leg infection. Approximately one year prior to presentation, she sustained a right distal femur fracture treated with retrograde intramedullary nail fixation, which was complicated by early hardware failure requiring revision. She then developed a MSSA infection, requiring removal of hardware and placement of an antibiotic spacer. She completed a course of antibiotics and subsequently underwent total knee arthroplasty, which was complicated by MRSA infection with development of a sinus tract communicating with the distal femur. She underwent three more irrigation and debridement procedures, long-term IV Vancomycin, and replacement of an antibiotic spacer at the most recent debridement. Her sinus tract persisted and she presented for second opinion to discuss alternatives to amputation. On examination, she had a draining sinus tract near the knee, which probed to a depth of 5 cm. Her right lower extremity was 4 cm shorter than the left. ESR was 16 mm/hr and CRP 0.58 mg/l.

**Figure 9:**
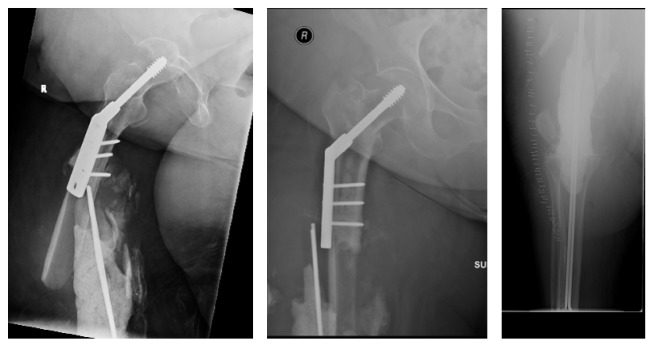
AP and lateral radiographs of right hip and lateral radiograph of right knee at presentation. Radiographs with antibiotic spacer over intramedullary K wire in tibia and extramedullary in femur. DHS in proximal femur.

**Figure 10:**
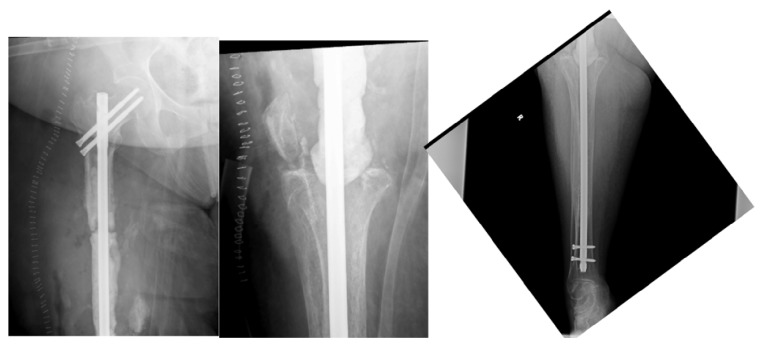
Postoperative radiographs showing long IM nail and surrounding bone cement.

Radiographs at presentation are shown (Figure 9), and she was taken to the operating room where cultures were obtained and all hardware and antibiotic cement were removed. The femur and tibia were then reamed and an 11.5 mm × 700 mm Smith and Nephew intramedullary nail was placed with gentamicin cement packed around the nail. Intraoperative cultures had no growth and she was placed on IV Vancomycin for six weeks given her history of MRSA and MSSA. Postoperative images are shown (Figure 10).

Her postoperative course was complicated by Vancomycin-resistant* Enterococcus* (VRE) PICC line infection and* E. coli* UTI, which were treated at an outside hospital. At follow-up, she was noted to have a draining sinus at the distal femur, which was treated with wet-to-dry dressings. Inflammatory markers were then trended and decreased over time off antibiotics.

**Figure 11:**
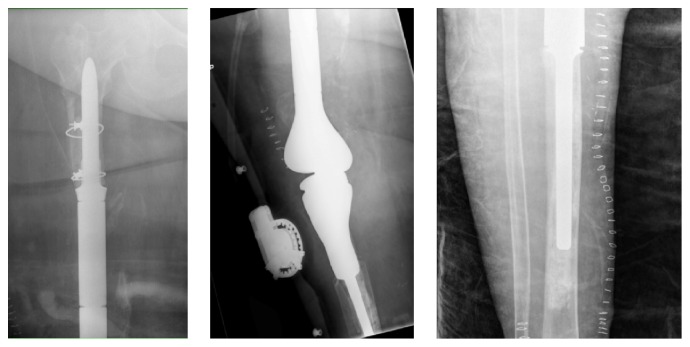
Postoperative radiographs of large endoprosthesis reconstruction.

Ten months later, it was felt that her infection had been cleared when her inflammatory markers reached ESR 10 mm/hr and CRP 0.26 mg/l, and she returned to the operating room for reconstruction. Intraoperatively, frozen sections were negative for acute inflammation and a large Stryker GMRS endoprosthesis was used for reconstruction. A gastrocnemius flap and split thickness skin graft were used for soft tissue coverage. Postoperative radiographs are shown (Figure 11).

Her postoperative course was complicated by necrosis and purulence of the proximal portion of the wound requiring repeat IV antibiotics and irrigation and debridement on three subsequent occasions over several months. Operative cultures from these debridements grew various organisms including VRE,* Morganella morganii*,* Acinetobacter baumannii*, and coagulase-negative* Staphylococcus*. A long discussion was held with the patient about her postoperative course when she continued to have drainage from the incision and intermittent wound breakdown despite attempts at antibiotic suppression. She was taken again to the operating room for hip disarticulation two years after her index procedure.

## 3. Discussion

The most important factor for successful treatment of chronic osteomyelitis is adequate debridement [1, 5]. Even with adequate debridement, the long-term recurrence rate is approximately 20% [6] and many patients end up with amputations at some level [3]. In order to preserve the limb, the area of skeletal debridement/resection must be restored. Bioactive glass, osteocutaneous flaps, antibiotic-loaded hydroxyapatite [1, 7, 8], and Ilizarov distraction osteosynthesis [4, 9] are well described for smaller defects, while autograft, allograft, or vascularized fibular grafting may be used for larger defects. If the articular surface is involved, however, these techniques will likely result in arthrodesis of the knee [10–12]. Another surgical option is Van Ness rotationplasty; however, careful patient selection is required for this procedure [13].

Endoprosthetic reconstruction following debridement of chronic osteomyelitis involving the distal femur has been described in the literature but is not commonly used for management [14]. A two-stage procedure is advised with these patients, given the risk of residual infection of the soft tissue bed immediately following debridement. In our hands, stage one requires debridement and placement of an intramedullary nail encased in antibiotic cement spanning the bony defect. Intravenous and oral antibiotics are administered, followed by a “cooldown” period (off antibiotics) with the goal of assessing the likelihood of residual infection. Once the infection is believed to be resolved, stage two then requires reconstruction with a large, segmental endoprosthesis.

These three patients received six weeks of IV antibiotics and, in cases 1 and 2, an additional six weeks and six months of oral antibiotics, respectively. Currently, there is insufficient evidence to identify the optimum duration or type of antibiotics to use [6]. It is imperative that an infectious disease team be closely involved and that patients be counseled about the risk of recurrent infection.

What made case 3 unsuccessful remains unclear. This patient had a sinus tract, had previously failed attempted debridement and placement of an antibiotic spacer, and had more medical comorbidities than the other two patients. Additionally, she was the only patient not to receive both IV and oral antibiotics after stage 1 of surgery, and her infection was polymicrobial with several highly virulent organisms. Which of these factors was the most important in regard to her outcome is difficult to determine; likely each contributed to her failure. This patient was given a guarded prognosis for success prior to reconstruction; however, she was unwilling to undergo amputation and showed no evidence for persistent infection on preoperative laboratory analysis or on frozen histopathological assessment intraoperatively.

In the appropriate patient, counseled extensively on the long treatment course and potential complications (including possible amputation), the above represents another option for management of chronic osteomyelitis of the distal femur.
